# Case report: Gouty renal abscess and gouty sacroiliitis associated with genetic variants of a young woman

**DOI:** 10.3389/fmed.2025.1509018

**Published:** 2025-02-28

**Authors:** Dabin Tang, Yubao Jiang, Guichen Ling, Jianyong Zhang, Jingjing Xie

**Affiliations:** ^1^The Fourth Clinical Medical College of Guangzhou University of Chinese Medicine, Shenzhen, Guangdong, China; ^2^The Department of Rheumatology, Shenzhen Traditional Chinese Medicine Hospital, Shenzhen, Guangdong, China

**Keywords:** gout, renal abscess, sacroiliitis, genetic variants, young woman

## Abstract

Gout is a form of inflammatory arthritis characterized by the deposition of monosodium urate (MSU) crystals in the joints, resulting from a disorder in purine metabolism. It occurs more frequently in men than in women prior to menopause and is rare in young women. Gout can lead to various health complications, with many patients experiencing a significant burden of chronic kidney disease (CKD) and joint deformities. The development of gout is influenced by a complex interplay of genetic, environmental, and lifestyle factors, with elevated serum MSU levels serving as a key risk factor for its onset. However, only 10% of individuals with hyperuricemia go on to develop clinical gout, and several susceptibility loci are associated with the condition. Here, we present a case of a young woman with gouty sacroiliitis and gouty nephropathy linked to susceptibility loci.

## 1 Introduction

Gout is the most prevalent form of inflammatory arthritis, typically presenting as monoarthritis that predominantly affects peripheral joints, while involvement of the sacroiliac joints is rare (1). The condition is significantly more common in men, and its prevalence varies across different racial groups, ranging from approximately 0.003 to 15.3% (2). Gout is recognized as one of the most widespread metabolic disorders, with its prevalence on the rise, particularly in developing countries. This increase can be attributed to factors such as population aging, dietary and lifestyle changes, and rising levels of obesity and insulin resistance. Chronic gout imposes a considerable social and economic burden due to the associated pain and disability, as well as diminished work-related activity and productivity (3).

Patients diagnosed with gout have an increased likelihood of developing chronic kidney disease (CKD) stage 3 or higher compared to individuals without gout (4). This notable clinical association raises important concerns regarding patient management and outcomes. Several factors contribute to this relationship. Firstly, hyperuricemia can have a direct adverse impact on renal function. Elevated levels of uric acid can lead to the formation of monosodium urate crystals within the renal tubules, resulting in obstructive nephropathy and subsequent inflammation. This process can significantly impair renal function, leading to the progression of kidney disease. Moreover, the use of nonsteroidal anti-inflammatory drugs (NSAIDs) in the management of gout flares may introduce additional risk factors of renal toxicity. NSAIDs are effective for pain relief and inflammation control, they can adversely affect renal perfusion and glomerular filtration, particularly in patients with pre-existing renal dysfunction. This potential nephrotoxicity necessitates careful consideration in the treatment of gout, especially in those with damaged kidney function (4, 5).

The heritability of serum monosodium urate (MSU) concentrations is estimated to range between 40 and 70%. Previous genome-wide association studies (GWAS) have identified numerous genetic loci associated with elevated serum uric acid levels and the development of gout (6). Here we report a female patient who exhibits two susceptibility loci, specifically SLC2A9 and SLC22A12, both of which are implicated in the deposition of monosodium MSU crystals in the sacroiliac joint and the kidneys. This case highlights the necessity for further investigation into the genetic factors contributing to gout, particularly in individuals with identified risk alleles. Understanding the genetic background may provide valuable insights into personalized treatment strategies and preventive measures for patients at high risk of developing gout and related renal complications. Such an approach could enhance clinical outcomes and improve the management of this prevalent metabolic disorder.

## 2 Case presentation

A 31-year-old woman has a history of 8 years of alcohol consumption and 4 years of consuming fructose-based beverages, with infrequent intake of water. Five years ago, the patient experienced pain in the first metatarsophalangeal joints of the right foot, with a uric acid level of 461 μmol/L, diagnosis of gout was obtained. The patient suffered recurrent episodes of gouty arthritis by taking NSAIDs. Recently, she suffered recurrent lower back pain. One week prior to admission, the patient reported recurring pain localized to the left lumbar region and the bilateral lumbosacral area, accompanied by mild morning stiffness that proved resistant to analgesic treatment.

At the time of admission, the patient presented with anemia, mental fatigue, occasional shortness of breath, and dizziness. Additionally, she exhibited swelling and pain in the left knee joint, as well as discomfort in the left lumbar region and lumbosacral area, accompanied by mild morning stiffness in both elbows, knees, and ankles. Importantly, there were no reported symptoms of abdominal pain, acid reflux, or skin rash.

Laboratory examinations revealed a white blood cell count of 16.35 × 10^9/L, with a neutrophil percentage of 79.5%. Hemoglobin levels was 72 g/L, and platelet count was 344 × 10^9/L. Serum uric acid levels were significantly elevated 734 μmol/L, with creatinine levels at 182 μmol/L and serum albumin at 25.8 g/L. C-reactive protein was markedly elevated at 116 mg/L, while transferrin levels were low at 1.9 g/L. Ferritin levels were notably high at 1454 ng/mL, and the human leukocyte antigen (HLA)-B27 was found to be negative. Anti-nuclear antibody, anti-neutrophil cytoplasmic antibody, and vasculitis-related antibodies yielded negative results. Serological tests for tuberculosis, Lyme disease, and Brucella, along with serial blood cultures, all demonstrated negative findings.

**Figure 1:**
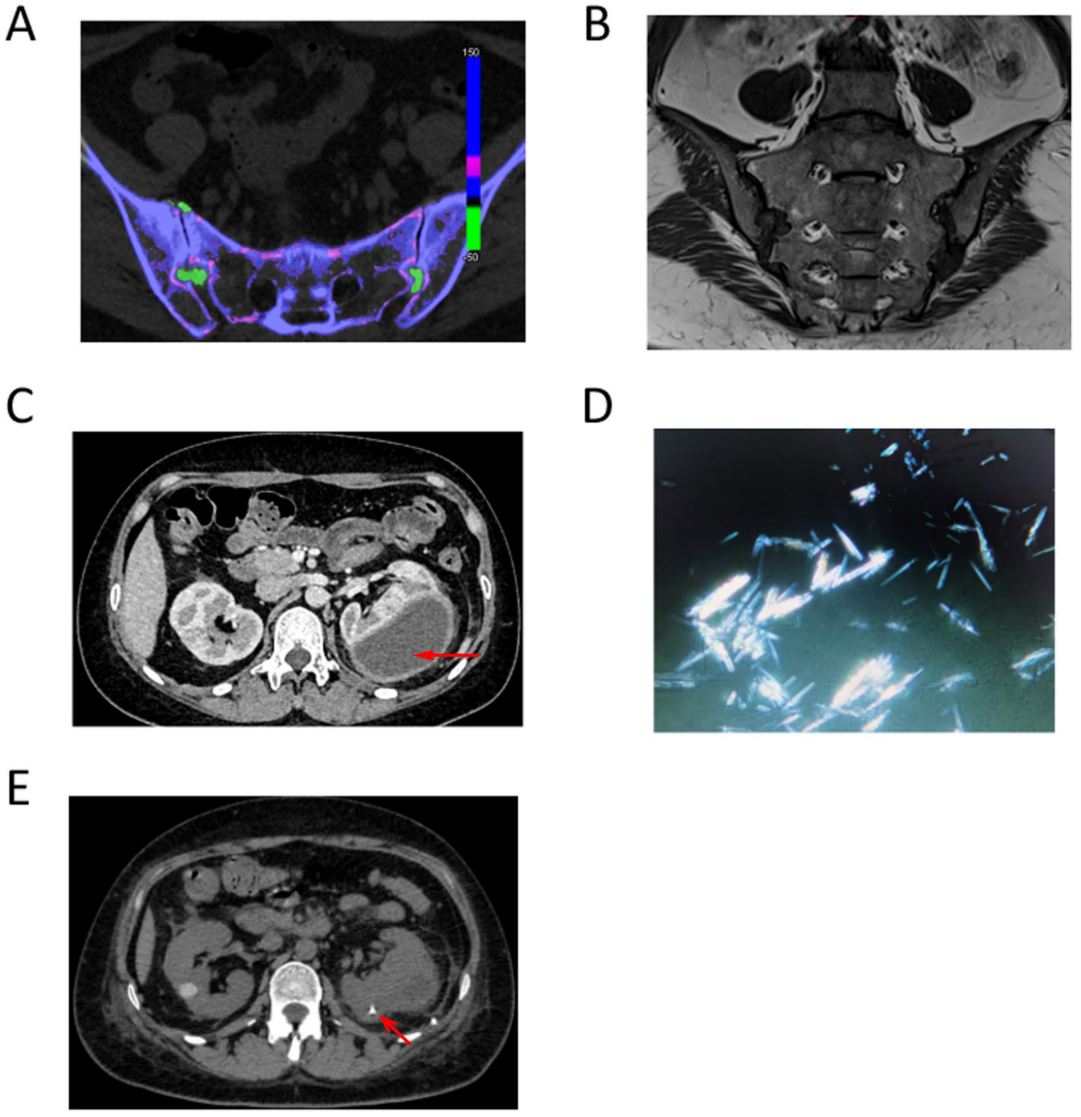
**(A)** Dual-source CT demonstrated a grass-green color change, and adjacent subarticular bony structures exhibited penetrating bone destruction. **(B)** Magnetic resonance imaging (MRI) of the sacroiliac joints revealed localized flaky long T1 and long T2 fat signal shadows beneath the joint surfaces of the bilateral tubular structures, accompanied by narrowing of the joint spaces and irregularities of the joint surfaces. **(C)** Enhanced CT scan review of abdomen indicated the left kidney was enlarged, presenting a mass-like low-density shadow in the left renal cortex measuring approximately 47 × 68 mm. **(D)** Polarized light microscopy revealed the presence of birefringent needle-like crystals. **(E)** Comparison of repeat CT indicated a newly observed catheter retention in the left kidney. The left renal cortex presented an irregular mass of low-density shadows, which has decreased in size since the previous examination, with slightly blurred borders. The maximum cross-sectional dimensions of the mass were approximately 22 mm × 38 mm.

Computed tomography (CT) of the sacroiliac joints revealed asymmetric narrowing of the bilateral sacral joint spaces, with patchy and nodular hyperdense shadows observed. Dual-source CT demonstrated a grass-green color change, and adjacent subarticular bony structures exhibited penetrating bone destruction, particularly in the iliac bone, where some edges displayed osteosclerotic changes (Figure 1A). Magnetic resonance imaging (MRI) of the sacroiliac joints revealed localized flaky long T1 and long T2 fat signal shadows beneath the joint surfaces of the bilateral tubular structures, accompanied by narrowing of the joint spaces and irregularities of the joint surfaces. No abnormal signals were detected within the sacral canal (Figure 1B). Enhanced CT scan review of abdomen indicated the left kidney was enlarged, presenting a mass-like low-density shadow in the left renal cortex measuring approximately 47 × 68 mm. This lesion exhibited clear borders and a CT value of approximately 20 HU, with no significant enhancement noted (Figure 1C, red arrow).

Given the possibility of a left renal abscess due to infection, a left renal puncture and aspiration were conducted, resulting in the extraction of approximately 120 mL of purulent and hemorrhagic fluid from the hematoma. Polarized light microscopy revealed the presence of birefringent needle-like crystals (Figure 1D). Biochemical analysis of the aspirated fluid indicated a total protein concentration of 27.8 g/L, with lactate dehydrogenase measured at 2870 U/L and glucose levels at 0.30 mmol/L. Serum protein electrophoresis demonstrated the following percentages: α2 globulin at 12.1%, albumin at 38.0%, α1 globulin at 4.4%, and *γ* globulin at 32.0%. Bacterial culture, blood immunofixation electrophoresis, and tuberculosis smears yielded no abnormalities. Following aspiration, an abdominal CT scan was performed again. Comparison of repeat CT indicated a reduction in the volume of the left kidney compared to previous imaging. A newly observed catheter retention was noted in the left kidney adjacent to the lumbar dorsal body (Figure 1E, red arrow). The left renal cortex presented an irregular mass of low-density shadows, which has decreased in size since the previous examination, with slightly blurred borders. The maximum cross-sectional dimensions of the mass were approximately 22 mm × 38 mm, and the CT value measured approximately 18 HU (Figure 1E).

Considering the patient’s status as a young woman with significant deposition of MSU crystals in the sacroiliac joints and kidneys, an investigation into the presence of susceptibility alleles was conducted through gene sequencing. Analysis of single nucleotide polymorphisms (SNPs) associated with MSU concentration revealed that the patient had a G/A genotype at the rs3733591 locus in the SLC2A9 gene (with allele G identified as a risk variant) and a C/C genotype at the rs893006 locus in the SLC22A12 gene (with allele C also recognized as a risk variant). Consequently, it is plausible that the patient carries risk alleles that may have been exacerbated by a prolonged high-purine diet, leading to the deposition of uric acid crystals in multiple anatomical sites. In the follow-up treatment, prednisone and colchicine were administered to control acute inflammation. Following the remission, a regimen of colchicine at 1 mg and febuxostat at 40 mg daily was initiated as therapeutic measurements.

## 3 Discussion

Gout is the most prevalent form of inflammatory arthritis in adult males, while it seldom occured in young females, suggesting that hormonal differences may play a critical role in the varying prevalence of gout between genders (7). Estrogen can enhance uric acid excretion through the kidneys, which helps maintain lower serum uric acid levels in premenopausal women. This protective effect diminishes after menopause, leading to an increased risk of gout in older women (8, 9). Several lifestyle and metabolic factors contribute to the onset of gout, and these can differ between men and women. Men are more likely to engage in behaviors that increase these risks, such as higher alcohol intake and diets rich in purines, found in red meat and certain seafood (10). In this case, despite being a young woman, she had a prolonged history of excessive consumption of alcohol and sugary beverages, leading to increased production of uric acid, which was a significant factor contributing to the onset of gout.

Sacroiliitis is characterized by recurrent lumbosacral pain and may be associated with conditions such as ankylosing spondylitis, inflammatory bowel disease, Crohn’s disease, tuberculosis, brucellosis, and osteoarthritis. While gout predominantly affects the first metatarsophalangeal joints, the sacroiliac joints may be involved in rare cases, closely resembling axial spondyloarthropathies or infections (11, 12). CT-guided needle aspiration of the affected joint remains the gold standard for diagnosing gout, arthrocentesis can be technically challenging at difficult-to-access sites, such as sacroiliac joint, and in patients with minimal joint effusion. In such cases, DECT can be a useful tool because it can specifically identify MSU crystal deposition based on its chemical composition. DECT examinations of gout frequently reveal penetrating chisel-like bone destruction, which may be represented as the “edge of uvula” sign. The presence of bone destruction located distant from the articular surface is a distinguishing characteristic that differentiates gouty arthritis from other forms of arthritis. Therefore, DECT has a high sensitivity and specificity in identifying MSU crystals of size >3 mm, with an overall accuracy of 87–94% (13, 14). In this case, DECT clearly demonstrated a substantial accumulation of MSU crystals in the sacroiliac joints. To eliminate the possibility of false positives, MRI was subsequently employed to further verify the presence of abnormal substances deposited in the sacroiliac joints. This finding, in conjunction with the patient’s clinical history, facilitated the conclusion that the recurrent lumbosacral pain was attributable to inflammation caused by MSU crystals.

MSU crystals can accumulate in the kidneys, resulting in gouty nephropathy and potentially leading to uremia. In the United Kingdom, patients with incident gout from a primary care population demonstrated a 78% increased risk of developing CKD stage 3 within three years of the gout diagnosis (4). The pathophysiological mechanisms by which hyperuricemia induces renal damage are complex. Uric acid can crystallize within the renal tubules, resulting in obstructive nephropathy. These crystals may trigger an inflammatory response, leading to tubulointerstitial fibrosis, characterized by scarring and a decline in kidney function (5). In this case, the patient presented with multiple cysts in both kidneys, within which MSU crystals were deposited, culminating in the formation of a substantial subperitoneal pus cavity that mimicked a renal infection. This condition was ultimately determined to be caused by MSU crystals, as clarified through nephrocentesis drainage.

Gout is a metabolic disorder associated with genetic predisposition, the primary mechanism involves the genes responsible for uric acid metabolism, particularly those coding for enzymes and transporters involved in its synthesis and excretion (6, 15). One key player is the SLC2A9 gene, which encodes glucose transporter 9 (GLUT9, also referred to as GLUT9L), plays a pivotal role as a uric acid transporter, facilitating the reabsorption of uric acid in the kidneys (16). The SLC2A9 gene has two primary transcript variants. The longer isoform of SLC2A9 is expressed on the basolateral membrane of proximal renal tubular epithelial cells, and its loss of function results in hypouricemia. Conversely, the shorter isoform is localized to the apical membrane of proximal renal tubular cells and is believed to mediate the absorption of urate from the renal tubule (17). Missense variants in SLC2A9 have demonstrated a significant association with clinical gout across diverse populations (18–20). The impact of variation in SLC2A9 appears to be more pronounced in females, accounting for approximately 6% of the variance in serum urate levels, compared to 2% in males (21, 22). Furthermore, SLC2A9 expression has been identified in human articular chondrocytes, a key site of urate deposition in gout. Synovial tissue has also been shown to express urate transporters. However, the extent to which urate transport is coordinated and regulated remains unclear. Investigating this mechanism is of critical interest for understanding the pathogenesis of tophus formation in gout.

Another significant gene associated with hyperuricemia and clinical gout is SLC22A12, which encodes the transporter protein urate transporter-1 (URAT1). URAT1 was the first confirmed urate transporter and is primarily responsible for the reabsorption of urate following urine filtration (23). It is predominantly expressed in the proximal tubules of the kidney, where it plays a critical role in maintaining uric acid homeostasis. By mediating uric acid reabsorption, SLC22A12 significantly influences serum uric acid levels (24). Variants in this gene can result in altered uric acid handling and subsequently impact an individual’s susceptibility to developing gout. Associations between variations in SLC22A12 and clinical gout have been demonstrated across diverse populations (25). Therapeutic interventions targeting URAT1 have proven effective in lowering uric acid levels, such as probenecid and benzbromarone. Additionally, a novel URAT1 inhibitor, lesinurad (marketed as Zurampic® or RDEA594), was approved in the United States and Europe in 2016 for the treatment of chronic gout (26). However, monotherapy with lesinurad has been associated with renal impairment, necessitating close monitoring of renal function in recipients (27). Research has identified various single nucleotide polymorphisms (SNPs) in the SLC2A9 and SLC22A12 gene that are associated with an increased risk of hyperuricaemia and gout, such as G/A genotype for rs3733591 in the SLC2A9 gene and a C/C genotype for rs893006 in the SLC22A12 gene. These variations can impair the kidney’s ability to excrete uric acid efficiently, resulting in developing hyperuricaemia and gout (28, 29). In this case, the young woman possesses a G/A genotype at the rs3733591 locus and a C/C genotype at the rs893006 locus, both of which significantly contribute to the development of gout. Therefore, it is essential to perform gene sequencing to identify at-risk individuals with gout, enabling better preventative measures against this disorder, particularly for young female patients.

One limitation of this case report is that we conducted dual-source CT imaging solely for sacroiliac joint, which confirmed the presence of MSU deposits. However, other affected joints, such as the knees and ankles, which also once exhibited swelling and pain, were not examined using dual-source CT, leaving the presence of MSU deposits in these areas unverified. Another limitation is that the patient presented with moderate anemia, but gastroenteroscopy ruled out gastrointestinal bleeding, and anemia-related diagnostic screenings did not reveal any abnormalities. The etiology of the anemia remains uncertain, with a possible link to renal blood leakage, potentially forming a renal hematoma.

In conclusion, gout is influenced by a complex interplay of genetic, environmental, and lifestyle factors. While dietary habits and metabolic conditions significantly contribute to its development, genetic susceptibility plays a critical role in an individual’s risk of developing gout and associated disorders.

## Data Availability

The raw data supporting the conclusions of this article will be made available by the authors, without undue reservation.

## References

[1] Malawista SE Seegmiller JE Hathaway BE Sokoloff L. Sacroiliac Gout. JAMA. (1965) 194:954–6. doi: 10.1001/jama.1965.03090220010002 5897962

[2] Singh JA Gaffo A. Gout epidemiology and comorbidities. Semin Arthritis Rheum. (2020) 50:S11–6. doi: 10.1016/j.semarthrit.2020.04.008, PMID: 32620196

[3] Smith E Hoy D Cross M Merriman TR Vos T Buchbinder R. The global burden of gout: estimates from the global burden of disease 2010 study. Ann Rheum Dis. (2014) 73:1470–6. doi: 10.1136/annrheumdis-2013-204647, PMID: 24590182

[4] Roughley M Sultan AA Clarson L Muller S Whittle R Belcher J. Risk of chronic kidney disease in patients with gout and the impact of urate lowering therapy: a population-based cohort study. Arthritis Res Ther. (2018) 20:243. doi: 10.1186/s13075-018-1746-1, PMID: 30376864 PMC6235219

[5] Kang DH Nakagawa T Feng L Watanabe S Han L Mazzali M. A role for uric acid in the progression of renal disease. J Am Soc Nephrol. (2002) 13:2888–97. doi: 10.1097/01.asn.0000034910.58454.fd 12444207

[6] Reginato AM Mount DB Yang I Choi HK. The genetics of Hyperuricaemia and gout. Nat Rev Rheumatol. (2012) 8:610–21. doi: 10.1038/nrrheum.2012.144, PMID: 22945592 PMC3645862

[7] Hak AE Choi HK. Menopause, postmenopausal hormone use and serum uric acid levels in us women – the third National Health and nutrition examination survey. Arthritis Res Ther. (2008) 10:R116. doi: 10.1186/ar2519, PMID: 18822120 PMC2592803

[8] Eun Y Kim IY Han K Lee KN Lee DY Shin DW. Association between female reproductive factors and gout: a Nationwide population-based cohort study of 1 million postmenopausal women. Arthritis Res Ther. (2021) 23:304. doi: 10.1186/s13075-021-02701-w, PMID: 34915918 PMC8675498

[9] Liu L Zhao T Shan L Cao L Zhu X Xue Y. Estradiol regulates intestinal Abcg2 to promote urate excretion via the Pi3k/Akt pathway. Nutrition Metabolism. (2021) 18:63. doi: 10.1186/s12986-021-00583-y, PMID: 34144706 PMC8212495

[10] Dehlin M Jacobsson L Roddy E. Global epidemiology of gout: prevalence, incidence, treatment patterns and risk factors. Nat Rev Rheumatol. (2020) 16:380–90. doi: 10.1038/s41584-020-0441-1, PMID: 32541923

[11] Guo QH Lu JG Zheng BL Li T Chen SH. Gouty Sacroiliitis: a case report of an often-overlooked cause of inflammatory Back pain. Int J Rheum Dis. (2023) 26:151–3. doi: 10.1111/1756-185X.14438, PMID: 36135353

[12] Baronio M Sadia H Paolacci S Prestamburgo D Miotti D Guardamagna VA. Etiopathogenesis of Sacroiliitis: implications for assessment and management. Korean J Pain. (2020) 33:294–304. doi: 10.3344/kjp.2020.33.4.294, PMID: 32989194 PMC7532300

[13] Bongartz T Glazebrook KN Kavros SJ Murthy NS Merry SP Franz WB 3rd. Dual-energy Ct for the diagnosis of gout: an accuracy and diagnostic yield study. Ann Rheum Dis. (2015) 74:1072–7. doi: 10.1136/annrheumdis-2013-205095, PMID: 24671771 PMC4431329

[14] Ogdie A Taylor WJ Weatherall M Fransen J Jansen TL Neogi T. Imaging modalities for the classification of gout: systematic literature review and Meta-analysis. Ann Rheum Dis. (2015) 74:1868–74. doi: 10.1136/annrheumdis-2014-205431, PMID: 24915980 PMC4869978

[15] Krishnan E Lessov-Schlaggar CN Krasnow RE Swan GE. Nature versus nurture in gout: a twin study. Am J Med. (2012) 125:499–504. doi: 10.1016/j.amjmed.2011.11.010, PMID: 22365026

[16] Vitart V Rudan I Hayward C Gray NK Floyd J Palmer CN. Slc2a9 is a newly identified urate transporter influencing serum urate concentration, Urate Excretion and Gout. Nat Genet. (2008) 40:437–42. doi: 10.1038/ng.106, PMID: 18327257

[17] Augustin R Carayannopoulos MO Dowd LO Phay JE Moley JF Moley KH. Identification and characterization of human glucose transporter-like Protein-9 (Glut9): alternative splicing alters trafficking. J Biol Chem. (2004) 279:16229–36. doi: 10.1074/jbc.M312226200, PMID: 14739288

[18] Hollis-Moffatt JE Xu X Dalbeth N Merriman ME Topless R Waddell C. Role of the urate transporter Slc2a9 gene in susceptibility to gout in New Zealand Māori, Pacific Island, and Caucasian case-control sample sets. Arthritis Rheum. (2009) 60:3485–92. doi: 10.1002/art.24938, PMID: 19877038

[19] Scharpf RB Mireles L Yang Q Köttgen A Ruczinski I Susztak K. Copy number polymorphisms near Slc2a9 are associated with serum uric acid concentrations. BMC Genet. (2014) 15:81. doi: 10.1186/1471-2156-15-81, PMID: 25007794 PMC4118309

[20] Dehghan A Köttgen A Yang Q Hwang SJ Kao WL Rivadeneira F. Association of Three Genetic Loci with uric acid concentration and risk of gout: a genome-wide association study. Lancet. (2008) 372:1953–61. doi: 10.1016/s0140-6736(08)61343-4, PMID: 18834626 PMC2803340

[21] Döring A Gieger C Mehta D Gohlke H Prokisch H Coassin S. Slc2a9 influences uric acid concentrations with pronounced sex-specific effects. Nat Genet. (2008) 40:430–6. doi: 10.1038/ng.107, PMID: 18327256

[22] Kolz M Johnson T Sanna S Teumer A Vitart V Perola M. Meta-analysis of 28, 141 individuals identifies common variants within five new loci that influence uric acid concentrations. PLoS Genet. (2009) 5:e1000504. doi: 10.1371/journal.pgen.1000504, PMID: 19503597 PMC2683940

[23] Enomoto A Kimura H Chairoungdua A Shigeta Y Jutabha P Cha SH. Molecular identification of a renal urate anion exchanger that regulates blood urate levels. Nature. (2002) 417:447–52. doi: 10.1038/nature742, PMID: 12024214

[24] Manolescu AR Augustin R Moley K Cheeseman C. A highly conserved hydrophobic motif in the exofacial vestibule of fructose transporting Slc2a proteins acts as a critical determinant of their substrate selectivity. Mol Membr Biol. (2007) 24:455–63. doi: 10.1080/09687680701298143, PMID: 17710649

[25] Vázquez-Mellado J Jiménez-Vaca AL Cuevas-Covarrubias S Alvarado-Romano V Pozo-Molina G Burgos-Vargas R. Molecular analysis of the Slc22a12 (Urat1) gene in patients with primary gout. Rheumatology. (2007) 46:215–9. doi: 10.1093/rheumatology/kel205, PMID: 16837472

[26] Miner JN Tan PK Hyndman D Liu S Iverson C Nanavati P. Lesinurad, a novel, Oral compound for gout, acts to decrease serum uric acid through inhibition of urate transporters in the kidney. Arthritis Res Ther. (2016) 18:214. doi: 10.1186/s13075-016-1107-x 27716403 PMC5048659

[27] Dai Y Lee CH. Transport mechanism and structural pharmacology of human urate transporter Urat1. Cell Res. (2024) 34:776–87. doi: 10.1038/s41422-024-01023-1, PMID: 39245778 PMC11528023

[28] Tu HP Chen CJ Tovosia S Ko AM Lee CH Ou TT. Associations of a non-synonymous variant in Slc2a9 with gouty arthritis and uric acid levels in Han Chinese subjects and Solomon islanders. Ann Rheum Dis. (2010) 69:887–90. doi: 10.1136/ard.2009.113357, PMID: 19723617

[29] Guan M Zhang J Chen Y Liu W Kong N Zou H. High-resolution melting analysis for the rapid detection of an Intronic single nucleotide polymorphism in Slc22a12 in male patients with primary gout in China. Scand J Rheumatol. (2009) 38:276–81. doi: 10.1080/03009740802572483, PMID: 19306160

