# Supplementary material for: Case report: Gouty renal abscess and gouty sacroiliitis associated with genetic variants of a young woman
Source: Front Med (Lausanne). 2025 Feb 28;12:1509018. doi: 10.3389/fmed.2025.1509018 (PMC11906439; doi:10.3389/fmed.2025.1509018)
Supplement: Supplementary file 1 [file Supplementary_file_1.pdf]

CARE case report guidelines

CARE Checklist of information to include when writing a case report

(cc) BY-NC-ND

Topic	Item	Checklist item description	Reported on Line
Title	1	The diagnosis or intervention of primary focus followed by the words "case report"	Line 1-3
Key Words	2	2 to 5 key words that identify diagnoses or interventions in this case report, including "case report"	Line 25
Abstract (no references)	3a	Introduction: What is unique about this case and what does it add to the scientific literature?	Line 12-22
	3b	Main symptoms and/or important clinical findings	Line 67-105
	3c	The main diagnoses, therapeutic interventions, and outcomes	Line 106-109
	3d	Conclusion—What is the main "take-away" lesson(s) from this case?	Line 264-267
Introduction	4	One or two paragraphs summarizing why this case is unique (**may include references**)	Line 28-64
Patient Information	5a	De-identified patient specific information.	Line 67-70
	5b	Primary concerns and symptoms of the patient.	Line 71-75
	5c	Medical, family, and psycho-social history including relevant genetic information	Line 68-71
	5d	Relevant past interventions with outcomes	Line 71-73
Clinical Findings	6	Describe significant physical examination (PE) and important clinical findings.	Line 76-122
Timeline	7	Historical and current information from this episode of care organized as a timeline	Line 72-81 Figure1
Diagnostic Assessment	8a	Diagnostic testing (such as PE, laboratory testing, imaging, surveys).	Line 82-122 Figure1
	8b	Diagnostic challenges (such as access to testing, financial, or cultural)	Line 106-122
	8c	Diagnosis (including other diagnoses considered)	Line 106-108
	8d	Prognosis (such as staging in oncology) where applicable	Line 159-162
Therapeutic Intervention	9a	Types of therapeutic intervention (such as pharmacologic, surgical, preventive, self-care)	Line 107
	9b	Administration of therapeutic intervention (such as dosage, strength, duration)	Line 106-107
	9c	Changes in therapeutic intervention (with rationale)	Line 106-107
Follow-up and Outcomes	10a	Clinician and patient-assessed outcomes (if available)	Line 116-122
	10b	Important follow-up diagnostic and other test results	Line 159-162
	10c	Intervention adherence and tolerability (How was this assessed?)	Line 159-162
	10d	Adverse and unanticipated events	NA
Discussion	11a	A scientific discussion of the strengths AND limitations associated with this case report	Line 165-263
	11b	Discussion of the relevant medical literature **with references**.	Line 165-351
	11c	The scientific rationale for any conclusions (including assessment of possible causes)	Line 264-267
	11d	The primary "take-away" lessons of this case report (without references) in a one paragraph conclusion	Line 264-267
Patient Perspective	12	The patient should share their perspective in one to two paragraphs on the treatment(s) they received	NA
Informed Consent	13	Did the patient give informed consent? Please provide if requested	Yes ☑ No ☐
